# Bisphenol A disturbs metabolism of primary rat adipocytes without affecting adipokine secretion

**DOI:** 10.1007/s11356-021-12411-0

**Published:** 2021-01-14

**Authors:** Katarzyna Szkudelska, Monika Okulicz, Tomasz Szkudelski

**Affiliations:** Department of Animal Physiology, Biochemistry and Biostructure, Poznań University of Life Sciences, Wołyńska 35, 60-637 Poznań, Poland

**Keywords:** Lipogenesis, Lipolysis, Adipocytes, Bisphenol A

## Abstract

Bisphenol A (BPA) is an ubiquitous synthetic chemical exerting numerous adverse effects. Results of rodent studies show that BPA negatively affects adipose tissue. However, the short-term influence of this compound addressing adipocyte metabolism and adipokine secretion is unknown. In the present study, isolated rat adipocytes were exposed for 2 h to 1 and 10 nM BPA. Insulin-induced glucose conversion to lipids along with glucose transport was significantly increased in the presence of BPA. However, basal glucose conversion to lipids, glucose oxidation, and formation of lipids from acetate were unchanged in adipocytes incubated with BPA. It was also shown that BPA significantly increases lipolytic response of adipocytes to epinephrine. However, lipolysis stimulated by dibutyryl-cAMP (a direct activator of protein kinase A) and the antilipolytic action of insulin were not affected by BPA. Moreover, BPA did not influence leptin and adiponectin secretion from adipocytes. Our new results show that BPA is capable of disturbing processes related to lipid accumulation in isolated rat adipocytes. This is associated with the potentiation of insulin and epinephrine action. The effects of BPA appear already after short-term exposure to low doses of this compound. However, BPA fails to change adipokine secretion.

## Introduction

Bisphenol A (BPA) is an ubiquitous environmental contaminant. It is thought that human populations worldwide are continuously being exposed to BPA, which largely contributes to numerous health problems. Exposure to BPA is associated, among others, with the risk of Parkinson disease, cancer, cardiovascular diseases, and metabolic disorders (Wade et al. 2020). Results of many studies also suggest that BPA markedly contributes to the development of type 2 diabetes (Wade et al. 2020). Type 2 diabetes is a serious metabolic disease affecting about 5% of people worldwide. This disease is characterized by metabolic abnormalities and also impaired insulin secretion and action. It is well established that the risk of type 2 diabetes is markedly increased in the case of adipose tissue dysfunction and obesity (International Diabetes Federation 2019). In this context, it was shown that increased exposure of humans to BPA may increase the risk of obesity (Wu et al. 2020). It is also known that in some populations, increased BPA levels are strongly associated with abdominal obesity (Lehmler et al. 2018; Wu et al. 2020).

Moreover, some studies have shown that in humans with type 2 diabetes urinary BPA levels are increased (Lang et al. 2008). Apart from the adverse effects of BPA observed in humans with type 2 diabetes, recent research indicate that this compound may impair glucose homeostasis also in healthy people (Wang et al. 2019). Different mechanisms are involved in the diabetogenic properties of BPA. One of them is an impairment of insulin action on target tissues. It is well established that BPA disturbs insulin signaling and thereby may induce insulin resistance. Such an effect is mainly due to reduced phosphorylation of some proteins of the insulin signaling pathway. This is strongly associated with the reduction of insulin-induced glucose transport into the skeletal muscle (Mullainadhan et al. 2017; Wade et al. 2020). Given a large mass of the skeletal muscle, reduced intramuscular glucose transport is associated with elevated blood glucose levels (Blaak 2005). Aside from impaired insulin action, BPA was also shown to induce mitochondrial dysfunction in pancreatic β-cells. This effect contributes to diminished insulin secretion and to the gradual failure of the insulin-secreting cells (Wei et al. 2011). Moreover, BPA alters the expression of some genes in pancreatic β-cells, which is also associated with decreased insulin secretion (Akash et al. 2020). This detrimental influence of BPA on β-cells additionally worsens the whole-body glucose homeostasis. BPA is also capable of inducing epigenetic modifications, especially DNA methylation. Rodent studies have shown that exposure to BPA during pregnancy and lactation increases the risk of diabetes in the next generation (Ma et al. 2013). Moreover, maternal exposure to environmental doses of BPA was shown to induce reproductive dysfunction in offspring (Olukole et al. 2019).

BPA, due to its chemical structure, has estrogenic properties, binds with estrogen receptors and thereby may negatively influence reproductive processes. However, estrogen receptors undergo expression in different tissues and are well known to play a relevant role in the regulation of many other processes, including glucose homeostasis. It is suggested that many detrimental effects elicited by BPA are mediated via these receptors (Ma et al. 2013; Acconcia et al. 2015).

BPA has also lipophilic properties and thereby is preferentially accumulated in adipose tissue (Fernandez et al. 2007). This tissue stores energy in the form of lipids, and excessive adipocyte lipid accumulation is strongly associated with metabolic disorders, insulin resistance and type 2 diabetes (Zatterale et al. 2020). Aside from energy accumulation, adipose tissue secretes also multiple adipokines, which have essential regulatory functions. These adipokines have been implicated in the regulation of feeding behavior, energy expenditure, glucose homeostasis, and many more (Fietta and Delsante 2013). Results of rodent studies have shown that BPA disturbs the metabolism of adipose tissue and also affects blood adipokine levels (Angle et al. 2013; Akash et al. 2020; Haq et al. 2020). Moreover, studies in vitro, on 3T3-L1 cells, have provided evidence that long-term exposure to BPA promotes adipogenesis. This is associated with the upregulated expression of adipogenic markers and increased lipid accumulation. It was also found that BPA is capable of limiting insulin-induced glucose utilization by 3T3-L1 cells (Ariemma et al. 2016). Apart from disturbed adipogenesis of 3T3-L-1 cells, detrimental effects of BPA in these cells cover also pro-inflammatory action. It was shown that BPA induces inflammatory processes and increases the levels of inflammatory markers in 3T3-L1 adipocytes (Ariemma et al. 2016; De Filippis et al. 2018). In spite of these data, there is a lack of results addressing the short-term effects of BPA on the metabolism of adipocytes and adipokine release. The aim of the present study was to determine 2-h exposure of primary rat adipocytes to BPA on processes related to energy accumulation, i.e., lipogenesis and lipolysis. Moreover, the effects of this compound on leptin and adiponectin secretion were also explored.

## Materials and methods

### Animals

Thirty male Wistar rats weighing 250–300 g were used in the experiments. Animals were purchased from Mossakowski Medical Research Centre Polish Academy of Sciences in Warsaw, Poland. They were kept in cages in pairs and maintained at standard conditions—in an air-conditioned animal room with a 12:12 dark-light cycle, in a constant temperature of 21 °C, with ad libitum access to tap water and standard laboratory diet (Labofeed B, “Morawski”, Kcynia, Poland). According to Polish law, experiments did not require agreement of the local ethics committee, because tissues were collected after the death and no experiments on alive animals were performed.

### Adipocyte isolation

Rats were decapitated and epididymal adipose tissue was collected for adipocyte isolation. Cells were isolated according to the method described by Rodbell with some modifications (Rodbell 1964; Szkudelska et al. 2008). The fat tissue was rinsed with 0.9% NaCl, placed in a plastic flask, and cut down into pieces. Then, Krebs-Ringer buffer with 3 mM glucose and 1 mg/ml collagenase was added. The buffer was gassed before use with a mixture of O_2_ and CO_2_ (95% and 5%) and its pH was adjusted to 7.4. Isolation was performed at 37 °C for 60 min with gentle shaking. After this time, adipocytes were filtered using a nylon mesh. Then, the isolated cells were rinsed with the buffer containing no collagenase. Afterward, cells were placed in polystyrene tubes and were left for flotation. The aliquots of the freshly isolated cells with the buffer were then taken for the appropriate experiments. Each treatment in all experiments was performed in five repetitions and was repeated in three separate experiments.

### Lipogenesis, glucose transport and oxidation

The effects of BPA on basal, insulin-stimulated glucose, and acetate conversion to total lipids in the isolated adipocytes were studied. For this purpose, these suspensions containing 10^6^ cells/ml/tube were incubated in the Krebs-Ringer buffer containing 3 mM glucose, D-[U-^14^C]-glucose (0.25 μCi) in basic or insulin (10 nM)-stimulated conditions—without BPA (control incubations)—or in the presence of this compound. Acetate conversion to lipids was measured in the presence of [1-^14^C]-acetate (0.25 μCi) and without insulin. Adipocytes were exposed to 1 or 10 nM BPA for 2 h. Then, lipogenesis was stopped, 5 ml of cold Dole’s extraction mixture containing isopropanol-heptane-1 N H_2_SO_4_ (40:10:1) was added and tubes were mixed (Dole and Meinertz 1960). The tubes were shaken, and afterward 2 ml of water and 3 ml of heptane were added and tubes were mixed once again. Finally, the upper phase was placed into the vials with the scintillation cocktail OptiPhase HiSafe 3 (PerkinElmer), and the radioactivity of total lipids was measured by using β-counter.

The glucose transport into adipocytes was measured by using the non-metabolizable analogue (2-deoxy-D-glucose). First, suspensions containing 10^6^ cells/ml/tube were preincubated with 0.5 mM glucose without BPA or with 1 or 10 nM BPA for 10 min at 37 °C with gentle shaking. Then, insulin (10 nM) was added to all tubes. After the next 20 min of incubations, 2-deoxy-D-[1-^3^H]-glucose (1 μCi) was added and the tubes were incubated once again for 3 min. To terminate the reaction, 400 μl of ice-cold Krebs-Ringer buffer with 3 mM phloretin was used. For separation of the cell suspensions from the buffer, 400 μl of silicone oil was added and tubes were centrifuged. The upper phase with cell suspensions was transferred into the vials with the scintillation cocktail OptiPhase HiSafe 3 (PerkinElmer), and the radioactivity was measured using β-counter.

Glucose oxidation was assayed by measurement of CO_2_ released from adipocytes during metabolism. Suspensions containing 10^5^ cells/ml/tube were incubated in the Krebs-Ringer buffer containing 3 mM glucose, D-[U-^14^C]-glucose (0.25 μCi), 1 nM insulin in the presence or absence of BPA at concentrations 1 nM or 10 nM. In each tube, the blotting paper saturated with hyamine hydroxide was placed above the surface of the incubation medium with cells. The tubes were tightly sealed with rubber stoppers. After 2 h of incubation with gentle shaking at 37 °C, 200 μl of 1 N H_2_SO_4_ was added through the stopper using a syringe with a thin needle and tubes were allowed to incubate for a further 1 h. After this time, blotting papers with adsorbed CO_2_ were placed in vials with the scintillation cocktail OptiPhase HiSafe 3 (PerkinElmer) and the radioactivity was measured using β-counter.

### Lipolysis and antilipolysis

Lipolysis was studied in the Krebs-Ringer buffer containing 3 mM glucose. To study the effects of BPA on lipolysis, two lipolytic stimulators were used. Initially, suspensions containing 10^6^ cells/ml/tube were incubated in the presence of 0.5 μM epinephrine alone or epinephrine with 1 or 10 nM BPA. Moreover, epinephrine was replaced by 0.5 mM dibutyryl-cAMP (DB-cAMP). In this part of the study, adipocytes were exposed to DB-cAMP alone or in the combination with 1 or 10 nM BPA.

To study the effects of BPA on antilipolysis, the combination of epinephrine with insulin was used. Adipocytes were incubated with 0.5 μM epinephrine and 1 nM insulin without BPA or in the presence of 1 or 10 nM BPA.

In each case, fat cells were incubated in the final volume of 1 ml for 2 h.

After this time, adipocytes were removed and concentrations of glycerol released by the cells to the incubation medium were determined. The colorimetric Hantzsch condensation method was used to glycerol assay according to the description of Foster and Dunn (1973) with some modifications. Ten percent trichloroacetic acid instead of Al_2_O_3_ was used to precipitate proteins, and there was no KOH used due to the lack of necessity of triglyceride hydrolysis. In the method, glycerol with the participation of sodium m-periodate is oxidized to formaldehyde. Formaldehyde then condenses with ammonia and acetylacetone to give a colored, yellow product (3,5-diacetyl-1,4-dihydrolutidine), which was measured at 410 nm.

### Adipokine secretion

Suspensions containing 10^6^ cells/ml/tube were incubated in Krebs-Ringer buffer containing 5 mM glucose in the presence of 10 mM insulin or insulin and 1 or 10 nM BPA for 3 h at 37 °C with gentle shaking. After incubation, cells were aspirated and adipokines released to the buffer were measured by using ELISA (the enzyme-linked immunosorbent assay method). The kits from two manufacturers were used—respectively—Rat Adiponectin/Acrp30 DuoSet ELISA, 5 Plate (R&D SYSTEMS, Inc., Minneapolis, MN, USA) for adiponectin and Rat Leptin PENTASET (BioVendor, Laboratorni medicina a.s., Brno, Czech Republic) for leptin measurement. The assays were performed according to the manufacturer’s instruction.

### Adipocyte viability

To determine the effects of BPA on adipocyte viability, suspensions containing 10^6^ cells/ml/tube were incubated for 2 h in the Krebs-Ringer buffer containing 3 mM glucose without BPA or in the presence of 1 and 10 nM BPA. At the end of incubations, all cells were rinsed with the buffer without BPA and then were exposed for 1 h to 0.5 mg/ml MTT (3-(4,5-dimethylthiazol-2-yl)-2,5-diphenyltetrazolium bromide). After this time, isopropanol was added, tubes were shaken and centrifuged, and the absorbance of isopropanol was read at 560 nm (Plumb 2004).

### Statistical analysis

The obtained results were expressed as means ± SEM of 15 determinations from three separate experiments evaluated statistically by one-way ANOVA and Tukey’s multiple comparison test using the GraphPad Prism for Windows software (license no. GRA/3802/2015, La Jolla, CA, USA). Differences were considered significant at *p* < 0.05.

## Results

### Effects of BPA on lipogenesis, glucose transport and oxidation

**Fig. 1:**
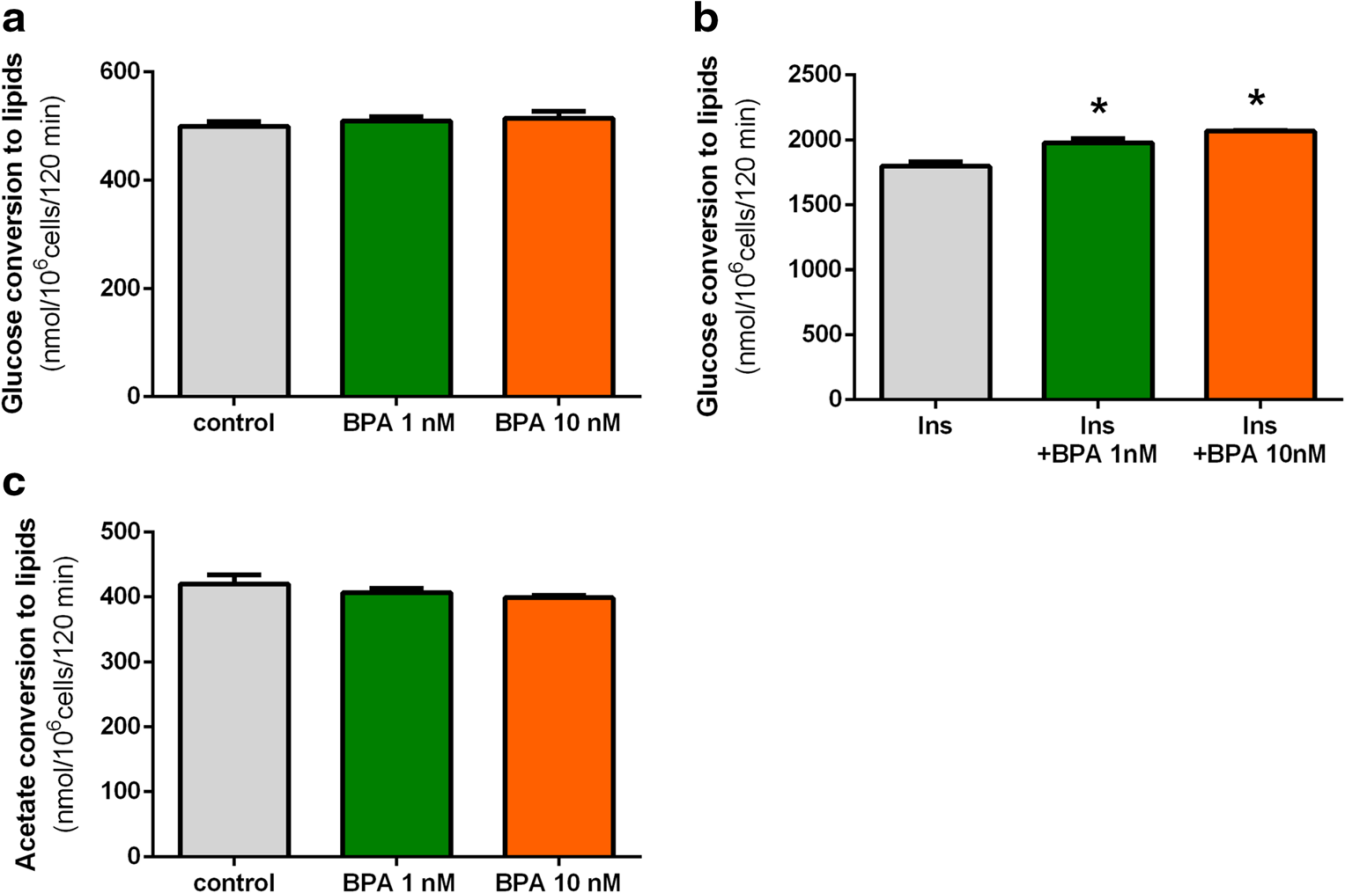
Effects of BPA on basal (**a**) and insulin-induced (**b**) lipogenesis from glucose (glucose conversion to lipids) and lipogenesis from acetate (acetate conversion to lipids) (**c**) in isolated rat adipocytes. To study lipogenesis from glucose, cells were incubated in the buffer containing unlabeled glucose and D-[U-^14^C]-glucose with insulin (Ins) (stimulated lipogenesis) or without this hormone (basal lipogenesis). Acetate conversion to lipids was studied in the buffer containing [1-^14^C]-acetate. Adipocytes were incubated without or with BPA. Values represent means ± SEM of 15 determinations from 3 separate experiments. Asterisk indicates differences statistically significant compared with incubations without BPA (*p* < 0.05)

In our present study, basal glucose conversion to lipids in the isolated rat adipocytes was not significantly affected by 1 and 10 nM BPA (Fig. 1a). However, insulin-stimulated glucose conversion to lipids in the isolated rat adipocytes was shown to be significantly (*p* < 0.05) increased in the presence of BPA (Fig. 1b). This effect was observed after 2-h exposure of adipocytes to BPA. The stimulatory effect was similar in the presence of 1 and 10 nM BPA and reached 12% and 15%, compared with lipogenesis in cells incubated without this compound (Fig. 1b).

Moreover, it was demonstrated that the formation of lipids from acetate was unchanged by the tested compound. In both cases, values observed in the presence of BPA were similar to those found during control incubations (Fig. 1c).

**Fig. 2:**
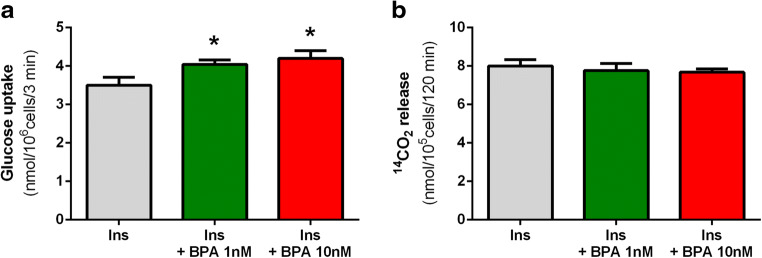
Effects of BPA on glucose transport (**a**) and oxidation (**b**) in isolated rat adipocytes. To study glucose transport (glucose uptake), adipocytes were preincubated in the presence of unlabeled glucose without or with BPA. Then insulin (Ins), and afterward 2-deoxy-D-[1-^3^H]-glucose were added to all tubes. To study glucose oxidation (determined as CO_2_ release), adipocytes were incubated in the buffer containing unlabeled glucose, D-[U-^14^C]-glucose, and insulin without or with BPA. Values represent means ± SEM of 15 determinations from 3 separate experiments. Asterisk indicates differences statistically significant compared with incubations without BPA (*p* < 0.05)

However, exposure of freshly isolated rat adipocytes to BPA allowed us to observe that this compound influences intracellular glucose transport. In the present study, insulin-induced glucose uptake was significantly (*p* < 0.05) augmented in adipocytes incubated for 2 h with 1 and 10 nM BPA, compared with the control cells. BPA was shown to increase this process by 16% and 20%, compared with the adipocytes exposed to insulin alone (Fig. 2a). Additionally, we also determined the effects of BPA on CO_2_ release from adipocytes. It was shown that the amount of CO_2_ release was irrespective of the presence of BPA, which indicates that this compound does not significantly change the glucose oxidation in adipocytes (Fig. 2b).

### Effects of BPA on lipolysis and antilipolysis

**Fig. 3:**
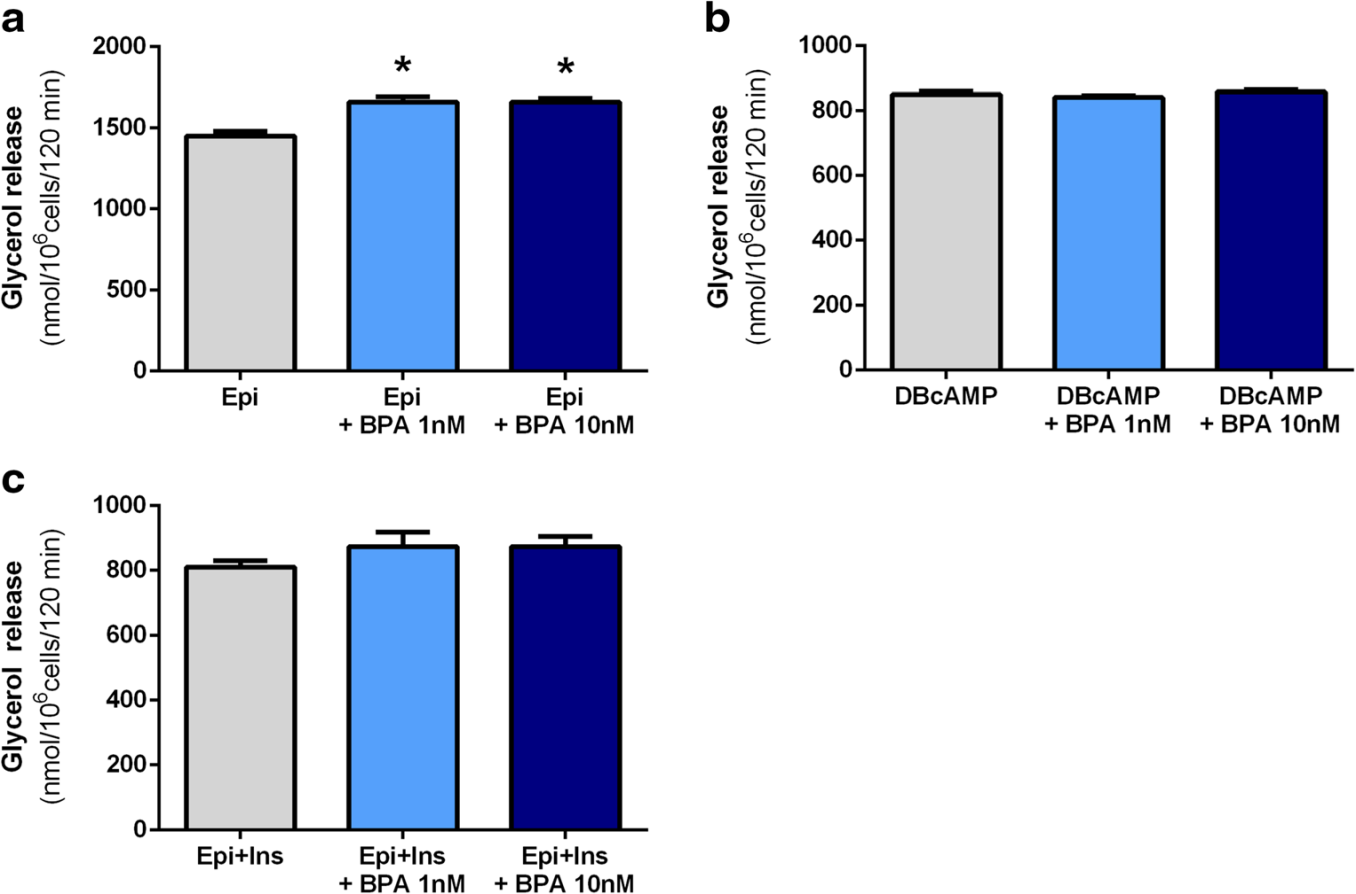
Effects of BPA on epinephrine (**a**)- and dibutyryl-cAMP (**b**)-induced lipolysis (determined as glycerol release) and on the antilipolytic action of insulin (**c**) in isolated rat adipocytes. Cells were incubated in the buffer containing epinephrine (Epi) (**a**), dibutyryl-cAMP (DB-cAMP) (**b**), or epinephrine and insulin (Ins) (**c**). Adipocytes were incubated without or with BPA. Values represent means ± SEM of 15 determinations from 3 separate experiments. Asterisk indicates differences statistically significant compared with incubations without BPA (*p* < 0.05)

Comparing the effects of BPA on epinephrine-induced lipolysis in the freshly isolated rat adipocytes, the significant changes induced by this compound were found. It was shown that the lipolytic response of adipocytes to epinephrine was significantly (*p* < 0.05) increased in the cells incubated for 2 h with 1 and 10 nM BPA. The glycerol release in the presence of 1 and 10 nM BPA was increased in both cases by 15%, compared with the effects elicited by epinephrine alone (Fig. 3a). However, lipolysis stimulated by DB-cAMP, a direct activator of PKA, was unaltered by BPA (Fig. 3b).

Additionally, the effects of BPA on the antilipolytic action of insulin were studied. It was demonstrated that lipolysis induced by epinephrine was reduced by about half in the presence of insulin. However, this antilipolytic action of insulin was not changed in adipocytes exposed for 2 h to 1 or 10 nM BPA (Fig. 3c).

### Effects of BPA on adipokine secretion

**Fig. 4:**
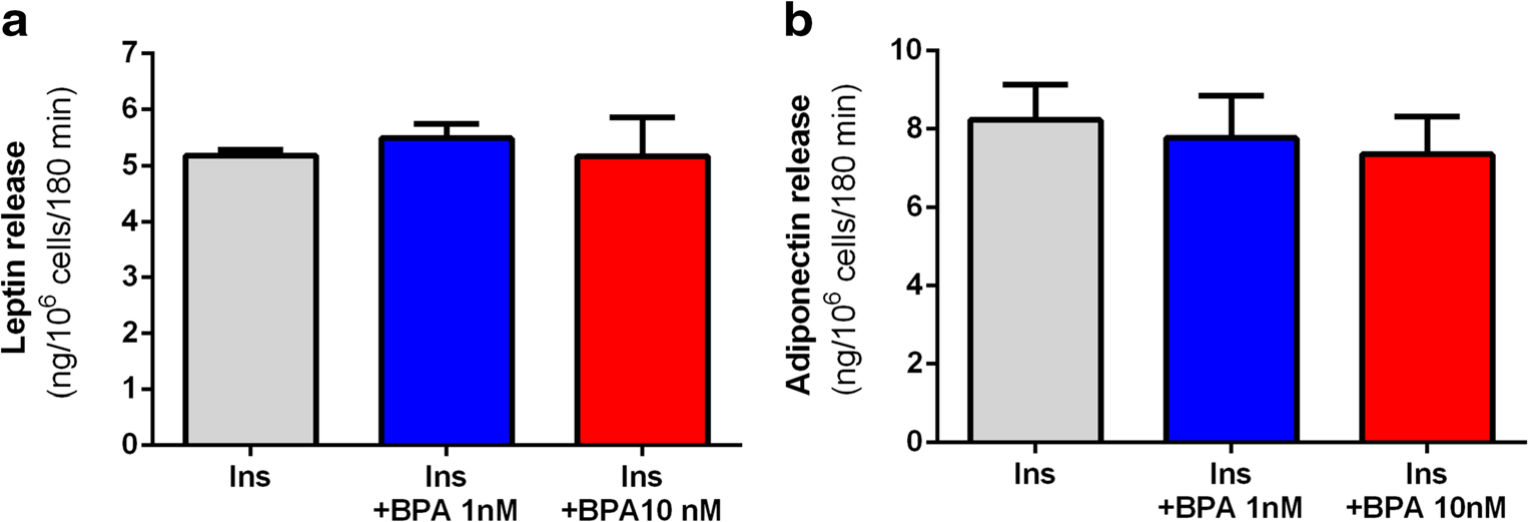
Effects of BPA on leptin (**a**) and adiponectin (**b**) release from isolated rat adipocytes. Cells were incubated in the buffer containing glucose and insulin (Ins). Adipocytes were incubated without or with BPA. Values represent means ± SEM of 15 determinations from 3 separate experiments

In the present study, leptin release from adipocytes incubated for 3 h with 1 and 10 nM BPA or without this compound did not differ significantly (Fig. 4a). Moreover, the results of our study also demonstrate that adiponectin release from freshly isolated rat adipocytes was not significantly affected as a result of exposure for 3 h to 1 and 10 nM BPA (Fig. 4b).

### Effects of BPA on adipocyte viability

It was shown that formazan formation from MTT did not significantly differ in adipocytes exposed for 3 h to 1 and 10 nM BPA or incubated without this compound. The mean absorbance was 0.530 ± 0.27, 0.546 ± 0.18, and 0.539 ± 0.41, respectively, in adipocytes incubated without BPA or in the presence of 1 and 10 nM BPA. This indicates that the tested compound does not affect adipocyte viability.

## Discussion

The results of the present study have revealed that BPA affects pivotal processes related to the accumulation of energy in the primary rat adipocytes, such as lipogenesis and lipolysis. It was shown that insulin-induced lipogenesis was significantly increased in the presence of BPA. Under physiological conditions, insulin is the main stimulator of lipogenesis. The glucose conversion to lipids promoted by this hormone covers many steps. The first one is intracellular glucose transport, followed by its metabolism and formation of triglycerides. The stimulatory effect of BPA on insulin-induced lipogenesis shown in the present study may result from different effects and may involve various steps of this process. Therefore, to better elucidate the action of BPA, its influence on glucose transport into adipocytes has been also explored. It was shown that, similarly to changes addressing lipogenesis, insulin-induced intracellular glucose transport was markedly increased in the presence of BPA, compared with fat cells incubated without this compound. Insulin stimulates glucose transport into adipocytes via glucose transporter GLUT4. In the presence of this hormone, glucose transport substantially increases, compared with non-stimulated cells. To elucidate whether the effects of BPA are dependent on insulin action, basal lipogenesis was also determined. This process was studied without insulin, when glucose reaches adipocytes by glucose transporter GLUT1. Additionally, the effects of BPA on lipogenesis from acetate were also compared. In this part of our experiment, glucose was replaced by acetate and adipocytes were incubated without insulin. Interestingly, it was shown that both basal glucose conversion to lipids and lipogenesis from acetate were not affected in cells exposed to BPA. These results clearly indicate that the stimulatory effect of BPA on insulin-induced lipogenesis is largely associated with the rise in glucose supply evoked by this hormone. It can be also concluded that BPA does not influence lipogenesis occurring without insulin. Other studies have shown that BPA increases the levels of GLUT4 in 3T3-F442A adipocytes, which is associated with increased intracellular lipid accumulation (Sakurai et al. 2004). This is in concert with our results indicating increased glucose transport in rat adipocytes after short-term exposure to BPA. These results strongly suggest that increased glucose transport via GLUT4 in the presence of BPA is a causative factor of enhanced lipogenesis.

Aside from glucose conversion to lipids, a part of this sugar undergoes also oxidation. This process could be also affected by BPA. Therefore, the effects of BPA on glucose oxidation in the presence of insulin were studied. It was, however, revealed that glucose oxidation was unchanged in the presence of BPA. This indicates that, among two essential pathways of glucose metabolism occurring in adipocytes, only lipogenesis is affected by BPA. Given that BPA stimulated glucose transport and its conversion to lipids, without affecting glucose oxidation, enhanced lipogenesis may be associated with increased activities of lipogenic enzymes.

The results of other research have shown that BPA decreases insulin-induced phosphorylation of protein kinase B (Akt), and also reduces glucose uptake stimulated by insulin in 3T3-L1 adipocytes. These effects were, however, observed after more prolonged incubations, compared with our study, and covered also changes in expression of some genes (Kidani et al. 2010; De Filippis et al. 2018). On the other hand, prolonged maintenance of 3T3-L1 cells with BPA may increase protein levels of GLUT4 with a concomitant decrease in glucose utilization (Ariemma et al. 2016). In our present study, the stimulatory effects of BPA on lipogenesis and glucose transport were found already after 2-h incubation. This clearly suggests that mechanisms other than changes in gene expression are implicated in the action of BPA in primary rat adipocytes. Moreover, it should be emphasized that all analyzed processes, such as lipogenesis, glucose transport, and glucose oxidation, were studied in the presence of insulin and were not decreased by BPA. This indicates that short-term effects of BPA on adipocytes are not associated with impaired insulin action. It was previously observed that a chronic exposure of 3T3-L1 cells to BPA increases lipid droplet accumulation in these cells (Ariemma et al. 2016). Lipogenesis in adipocytes is, however, a dynamic, hormone-regulated process. Therefore, our results show that, apart from long-term effects addressing gene expression or lipid droplet accumulation (Ariemma et al. 2016; De Filippis et al. 2018), BPA is also capable of inducing short-term changes in adipocytes, which cover the increase in insulin-stimulated lipogenesis and glucose transport.

Afterward, the effects of BPA on lipolysis in the primary rat adipocytes were also explored. Epinephrine-induced lipid breakdown was shown to be significantly increased in the presence of BPA. Epinephrine promotes lipolysis via a sequence of events involving the interaction with β-adrenergic receptor, followed by action of G_s_ protein, activation of adenylate cyclase, a rise in intracellular cyclic adenosine monophosphate (cAMP), activation of protein kinase A (PKA), stimulation of intracellular lipases, phosphorylation of perilipins, and finally triglyceride hydrolysis (Frühbeck et al. 2014). The influence of BPA on epinephrine-induced lipolysis may involve different steps of this pathway. To better elucidate its action, epinephrine was replaced by dibutyryl-cAMP (DB-cAMP). This is a direct activator of PKA, which acts with the omission of the steps occurring before this enzyme. It was shown that the lipolytic response of adipocytes to DB-cAMP was not significantly changed in the presence of BPA. This suggests that BPA enhances epinephrine-induced lipolysis affecting steps involving PKA or before. Other studies have reported that short-term exposure of rat cardiomyocytes (Gao et al. 2013) and human testicular seminoma cells (Bouskine et al. 2009) to 1 nM BPA evokes a rapid increase in cAMP content and activates PKA. Similar effects may be expected in adipocytes incubated with 1 or 10 nM BPA. Given that a rise in intracellular cAMP is largely associated with increased lipolysis (Frühbeck et al. 2014), this rise may be responsible for the potentiatory effect of BPA on epinephrine-induced lipolysis.

We also studied the effects of BPA on the antilipolytic action of insulin. Lipolysis was stimulated by epinephrine. As expected, epinephrine-induced lipid breakdown was markedly inhibited in the presence of insulin. The antilipolytic action of insulin mainly covers activation of phosphodiesterase 3B and the resulting decrease in intracellular cAMP (Frühbeck et al. 2014). Our results have shown that the antilipolytic action of insulin was not significantly altered in adipocytes subjected to BPA action, compared with cells incubated without this compound.

Aside from the effects of BPA on adipocyte metabolism, its influence on the endocrine functions of these cells was also studied. We focused on adiponectin and leptin secretion. These hormones are largely implicated in the regulation of numerous processes related to energy expenditure, glucose homeostasis, and many more. Leptin secretion undergoes short- and long-term regulation. The latter covers mainly changes in gene expression (Szkudelski 2007; Fietta and Delsante 2013). The previous studies with 3T3-L1 adipocytes have shown that long-term exposure of these cells to BPA may increase leptin gene expression without affecting the secretion of this hormone (Héliès-Toussaint et al. 2014; Ariemma et al. 2016). Our results have also shown that 3-h exposure of adipocytes to BPA failed to significantly change leptin secretion.

BPA was previously found to upregulate adiponectin gene expression in 3T3-L1 adipocytes (Ariemma et al. 2016). However, other studies on 3T3-L1 cells indicate that BPA is capable of reducing adiponectin production and secretion. These effects were observed at much higher concentrations of BPA and after exposure for 24 h (Kidani et al. 2010). The present study has shown that 3-h exposure of primary rat adipocytes to BPA is not associated with any significant changes in adiponectin secretion.

It is noteworthy that the concentrations of BPA used in our study were very low, i.e., 1 and 10 nM and comparable with environmental exposure (Akash et al. 2020; Wade et al. 2020). Results of in vitro studies indicate that BPA may elicit contrary effects depending on its concentrations (Héliès-Toussaint et al. 2014; Ramskov Tetzlaff et al. 2020; Wade et al. 2020). Our results have shown that the effects of this compound on adipocytes were not concentration-dependent, since similar changes were observed in the presence of 1 and 10 nM BPA. It can be also concluded that effects induced by BPA in rat adipocytes do not involve cellular damage. This assumption is associated with the use of low concentrations of BPA and was additionally confirmed by MTT test, showing that this compound does not affect adipocyte viability.

## Conclusion

Our new findings indicate that BPA increased the stimulatory effects of insulin on glucose conversion to lipids in the isolated rat adipocytes. This was accompanied by a simultaneous rise in insulin-induced glucose transport. These results imply that BPA did not impair the action of insulin. Moreover, BPA was shown to enhance the lipolytic response of adipocytes to epinephrine. The effects related to insulin and epinephrine indicate that BPA disturbs physiological regulation of adipocyte lipid accumulation. Importantly, all these changes were observed already after 2-h exposure of cells to BPA. It should be also emphasized that the effective concentrations of the tested compound used in our study were very small. The studies addressing adipokine release indicate a lack of BPA influence on the secretion of adiponectin and leptin. Our experiments were performed using freshly isolated rat adipocytes, which differ in many aspects from 3T3-L1 adipocytes. The use of various models may be the relevant reason for some differences in results.

## Data Availability

The datasets used and/or analyzed during the current study are available from the corresponding author on reasonable request.

## References

[CR1] Acconcia F, Pallottini V, Marino M (2015). Molecular mechanisms of action of BPA. Dose-Response.

[CR2] Akash MSH, Sabir S, Rehman K (2020). Bisphenol A-induced metabolic disorders: from exposure to mechanism of action. Environ Toxicol Pharmacol.

[CR3] Angle BM, Do RP, Ponzi D, Stahlhut RW, Drury BE, Nagel SC, Welshons WV, Besch-Williford CL, Palanza P, Parmigiani S, vom Saal FS, Taylor JA (2013). Metabolic disruption in male mice due to fetal exposure to low but not high doses of bisphenol A (BPA): evidence for effects on body weight, food intake, adipocytes, leptin, adiponectin, insulin and glucose regulation. Reprod Toxicol.

[CR4] Ariemma F, D'Esposito V, Liguoro D, Oriente F, Cabaro S, Liotti A, Cimmino I, Longo M, Beguinot F, Formisano P, Valentino R (2016). Low-dose bisphenol-A impairs adipogenesis and generates dysfunctional 3T3-L1 adipocytes. PLoS One.

[CR5] Blaak EE (2005). Metabolic fluxes in skeletal muscle in relation to obesity and insulin resistance. Best Pract Res Clin Endocrinol Metab.

[CR6] Bouskine A, Nebout M, Brücker-Davis F, Benahmed M, Fenichel P (2009). Low doses of bisphenol A promote human seminoma cell proliferation by activating PKA and PKG via a membrane G-protein-coupled estrogen receptor. Environ Health Perspect.

[CR7] De Filippis E, Li T, Rosen ED (2018). Exposure of adipocytes to bisphenol-A in vitro interferes with insulin action without enhancing adipogenesis. PLoS One.

[CR8] Dole VP, Meinertz H (1960). Microdetermination of long-chain fatty acids in plasma and tissues. J Biol Chem.

[CR9] Fernandez MF, Arrebola JP, Taoufiki J, Navalón A, Ballesteros O, Pulgar R, Vilchez JL, Olea N (2007). Bisphenol-A and chlorinated derivatives in adipose tissue of women. Reprod Toxicol.

[CR10] Fietta P, Delsante G (2013). Focus on adipokines. Theor Biol Forum.

[CR11] Foster LB, Dunn RT (1973). Stable reagents for determination of serum triglycerides by a colorimetric Hantzsch condensation method. Clin Chem.

[CR12] Frühbeck G, Méndez-Giménez L, Fernández-Formoso JA, Fernández S, Rodríguez A (2014). Regulation of adipocyte lipolysis. Nutr Res Rev.

[CR13] Gao X, Liang Q, Chen Y, Wang HS (2013). Molecular mechanisms underlying the rapid arrhythmogenic action of bisphenol A in female rat hearts. Endocrinology.

[CR14] Haq MEU, Akash MSH, Rehman K, Mahmood MH (2020). Chronic exposure of bisphenol A impairs carbohydrate and lipid metabolism by altering corresponding enzymatic and metabolic pathways. Environ Toxicol Pharmacol.

[CR15] Héliès-Toussaint C, Peyre L, Costanzo C, Chagnon MC, Rahmani R (2014). Is bisphenol S a safe substitute for bisphenol A in terms of metabolic function? An in vitro study. Toxicol Appl Pharmacol.

[CR16] International Diabetes Federation (2019) https://idf.org/

[CR17] Kidani T, Kamei S, Miyawaki J, Aizawa J, Sakayama K, Masuno HJ (2010). Bisphenol A downregulates Akt signaling and inhibits adiponectin production and secretion in 3T3-L1 adipocytes. Atheroscler Thromb.

[CR18] Lang IA, Galloway TS, Scarlett A, Henley WE, Depledge M, Wallace RB (2008). Association of urinary bisphenol A concentration with medical disorders and laboratory abnormalities in adults. JAMA.

[CR19] Lehmler HJ, Liu B, Gadogbe M, Bao W (2018). Exposure to bisphenol A, bisphenol F, and bisphenol S in U.S. adults and children. The National Health and Nutrition Examination Survey 2013–2014. ACS Omega.

[CR20] Ma Y, Xia W, Wang DQ, Wan YJ, Xu B, Chen X, Li YY, Xu SQ (2013). Hepatic DNA methylation modifications in early development of rats resulting from perinatal BPA exposure contribute to insulin resistance in adulthood. Diabetologia.

[CR21] Mullainadhan V, Viswanathan MP, Karundevi B (2017). Effect of Bisphenol-A (BPA) on insulin signal transduction and GLUT4 translocation in gastrocnemius muscle of adult male albino rat. Int J Biochem Cell Biol.

[CR22] Olukole SG, Lanipekun DO, Ola-Davies EO, Oke BO (2019). Maternal exposure to environmentally relevant doses of bisphenol A causes reproductive dysfunction in F1 adult male rats: protective role of melatonin. Environ Sci Pollut Res.

[CR23] Plumb JA (2004). Cell sensitivity assays: the MTT assay. Methods Mol Med.

[CR24] Ramskov Tetzlaff CN, Svingen T, Vinggaard AM, Rosenmai AK, Taxvig C (2020). Bisphenols B, E, F, and S and 4-cumylphenol induce lipid accumulation in mouse adipocytes similarly to bisphenol A. Environ Toxicol.

[CR25] Rodbell JM (1964). Metabolism of isolated fat cells. I. Effects of hormones on glucose metabolism and lipolysis. J Biol Chem.

[CR26] Sakurai K, Kawazuma M, Adachi T, Harigaya T, Saito Y, Hashimoto N, Mori C (2004). Bisphenol A affects glucose transport in mouse 3T3-F442A adipocytes. Br J Pharmacol.

[CR27] Szkudelska K, Nogowski L, Szkudelski T (2008). Genistein, a plant-derived isoflavone, counteracts the antilipolytic action of insulin in isolated rat adipocytes. J Steroid Biochem Mol Biol.

[CR28] Szkudelski T (2007). Intracellular mediators in regulation of leptin secretion from adipocytes. Physiol Res.

[CR29] Wade M, Delawder V, Reneau P, Dos Santos JM (2020). The effect of BPA exposure on insulin resistance and type 2 diabetes - the impact of muscle contraction. Med Hypotheses.

[CR30] Wang B, Li M, Zhao Z, Lu J, Chen Y, Xu Y, Xu M, Wang W, Wang T, Bi Y, Ning G (2019). Urinary bisphenol A concentration and glucose homeostasis in non-diabetic adults: a repeated-measures, longitudinal study. Diabetologia.

[CR31] Wei J, Lin Y, Li Y, Ying C, Chen J, Song L, Zhou Z, Lv Z, Xia W, Chen X, Xu S (2011). Perinatal exposure to bisphenol A at reference dose predisposes offspring to metabolic syndrome in adult rats on a high-fat diet. Endocrinology.

[CR32] Wu W, Li M, Liu A, Wu C, Li D, Deng Q, Zhang B, Du J, Gao X, Hong Y (2020). Bisphenol A and the risk of obesity - a systematic review with meta-analysis of the epidemiological evidence. Dose-Response.

[CR33] Zatterale F, Longo M, Naderi J, Raciti GA, Desiderio A, Miele C, Beguinot F (2020). Chronic adipose tissue inflammation linking obesity to insulin resistance and type 2 diabetes. Front Physiol.

